# Ultrafiltration of Black Soldier Fly (*Hermetia illucens*) and Mealworm (*Tenebrio molitor*) Protein Concentrates to Enhance Emulsifying and Foaming Properties

**DOI:** 10.3390/membranes13020137

**Published:** 2023-01-20

**Authors:** Madushika K. Ranasinghe, Aurélie Ballon, Sílvia de Lamo-Castellví, Montserrat Ferrando, Carme Güell

**Affiliations:** 1 Department d’Enginyeria Quimica, Universitat Rovira i Virgili, Avda. Països Catalans, 26, 43007 Tarragona, Spain; 2 Department of Animal Science, Faculty of Animal Science and Export Agriculture, Uva Wellassa University, Badulla 90000, Uva, Sri Lanka

**Keywords:** insect proteins, techno-functional properties, insect protein fractionation, regenerated cellulose acetate membrane

## Abstract

Mealworm, TM (*Tenebrio molitor*), and black soldier fly, BSF (*Hermetia illucens*) are of special interest for food and feed applications due to their environmental benefits such as low water and land requirements, low greenhouse gas emissions, and high feed-conversion efficiency. This study assesses the use of ultrafiltration (UF) to fractionate protein concentrates from TM and BSF (TMPC, BSFPC) in order to enhance emulsifying and foaming properties. A 30 kDa regenerated cellulose acetate membrane enabled the separation of concentrate and permeate fractions for both insect proteins from two different initial feed concentrations (10 and 7.5 g/L). Permeate flux and protein transmission behave differently depending on the insect type and the initial concentration; while for TMPC permeate flux increases with a decrease in the initial protein concentration, it is not affected for BSFPC. The existing membrane cleaning protocols are suitable for recovering water flux after UF of insect proteins, enabling membrane re-use. Emulsifying activity is maintained for all the TMPC fractions, but it is significantly lower for the permeate fractions of BSFPC. Foaming properties are maintained for all the UF fractions of BSFPC and the ones from 7.5 g/L TMPC. Acidic solubilization leads to a fraction with enhanced emulsifying capacity and one with higher foaming capacity than the original for BSFPC. This study opens the door to membrane technology for insect protein fractionation, which has not been studied so far and has already provided useful solutions for other animal and plant proteins.

## 1. Introduction

Because of the environmental benefits of insect-rearing for food and feed applications [1], there is an increasing need to develop suitable technologies to produce insect meals, fractionate the main ingredients, and use suitable processing technologies to yield high-quality, standardized, functional, and tailored insect fractions that can be incorporated into complex product formulations [2]. While the consumption of whole or minimally processed insects in western markets is still a matter of controversy (the “ick” factor), efficient processes for the recovery of functional insect-derived fractions such as protein, fat, or chitin are being developed to promote consumer acceptance, and to explore a wide range of potential food and non-food applications, with opportunities to use them in various forms being searched for [3]. 

Amongst the insect species that are currently under consideration, black soldier fly (*Hermetia illucens*) larvae are widely accepted for feed applications, while dried mealworm (*Tenebrio molitor*) has already received the favorable opinion of the European Food Safety Authority (EFSA) for food applications [4]. The insect protein content varies depending on the insect species, the development stage, the way they are processed (thermal, mechanical), and the protocol used for protein isolation. Black soldier fly (BSF) larvae contain 42% crude protein and 29% fat on average in dry matter [5], and although the protein content is lower than for other orthoptera species, the advantages of BSF are their survival rate and efficiency of converting organic materials into their own biomass. As for mealworm, it is considered as a good source of nutrients, with a protein content between 41–66%, fat content between 15–50%, fiber between 9–19% (all in dry bases), and high amounts of potassium, calcium, iron, and magnesium [6]. 

There is a growing interest in the potential of insect proteins as a source of functional and bioactive peptides. The existing knowledge of animal and plant proteins has guided research on the functional and bioactive potential of insect proteins and their hydrolysates. A review by Nongonierna et al. [7] presents the potential of insect protein hydrolysates and lists protein extraction and hydrolysis processes to produce bioactive peptides from several insect species, mainly silkworm (*Bombix mori*) but also mealworm, lesser mealworm (*Alphitobius diaperinus*), banded cricket (*Gryllodes sigillatus*), and migratory locust (*Locusta migratoria*). Enzymatic hydrolysis has been used to efficiently enhance bioactive properties such as antioxidant, antihypertensive, antidiabetic, and antimicrobial properties [8,9,10,11,12,13]. As for techno-functional properties of various insect powders and their protein extracts, including solubility, water and oil binding capacity, foaming capacity, surface hydrophobicity, gelling properties, and emulsifying ability, they have already shown promising outcomes [14,15,16,17,18,19]. Gkinali et al. have recently reviewed the potential of mealworm ingredients for the food industry [6]. They present an up-to-date summary of functional properties of mealworm flours, defatted flours, and protein extracts. Specifically for emulsifying properties, they conclude from the available literature, mealworm protein extracts have a similar ability to stabilize oil/water interfaces as commercial whey protein. As for foaming capacity, the results published so far indicated a good ability of defatted mealworm preparations for foam stabilization.

As far as techno-functional properties of BSF, Bußler et al. (2016) [17] presented one of the first studies on the effect of defatting on water- and oil-binding capacities, showing no significant changes between original and defatted samples. Recently, Mshayisa et al. [20] have reported the effect of different protein extraction methods on techno-functional properties of BSF extracts. They have compared alkaline extraction with alkaline extraction coupled with isoelectric point precipitation, finding that water-binding capacity (WBC) was the highest for BSF protein extracts obtained with alkaline extraction and isoelectric point precipitation, while the oil-binding capacity (OBC) was higher for the original flours (defatted and non-defatted) than for the protein extracts. Regarding emulsifying and foaming properties, they were enhanced in the protein extracts, regardless of the extraction method applied. Moreover, BSF protein concentrate has successfully been used to stabilize sunflower and lemon oil emulsions, produced using a high-throughput membrane emulsification system [5]. The authors showed that the distribution of droplet sizes for the emulsions and fluxes of emulsions prepared with BSF protein concentrates were comparable to the ones obtained with whey protein isolate, showcasing the potential of BSF to totally or partially replace whey protein in food formulations.

Regardless of the method used to obtain the protein fraction, there is no literature on fractionation of insect proteins with membrane technologies; therefore, the vast existing knowledge on membrane fractionation for dairy, fish, and plant proteins is highly relevant and the mandatory starting point for their design. Usually, whey proteins fractionation and purification by membrane filtration starts with a microfiltration (MF) or centrifugation for defatting and/or clarification. The permeate from MF can be subsequently fractionated using ultrafiltration (UF) to obtain concentrated fractions with a molecular weight range that will depend on the molecular weight cut-off (MWCO) of the membranes employed. A critical issue for insect protein fractionation by UF is the knowledge about their molecular weight distribution. Protein extracts from defatted BSF flours obtained after alkaline solubilization and precipitation at pH 4 presented two major bands at 14.3 and 80.5 kDa [17], while protein extracts from defatted mealworm flours isolated through the same process showed a different distribution of the protein fractions, with a major fraction between 13–40 kDa and another fraction between 67–250 kDa [17].

This work assesses for the first time the use of UF to fractionate mealworm and BSF protein concentrates to enhance interfacial properties such as foaming capacity, foam stability, and emulsifying activity. Based on previous knowledge of mealworm and BSF protein molecular size distribution, fractionation with a commercial regenerated cellulose acetate membrane of 30 kDa is suggested to obtain two protein fractions for each insect protein concentrate. The ability of both fractions to stabilize oil–water and air–water interfaces will be tested using standardized methods. The influence of the initial protein concentration in the flux and protein at the outlet of the filtration cell will be studied based on data from or after UF. Since there is no knowledge available, special attention will be paid to determining if the standard cleaning procedures provided by the manufacturer allow for the restoration of membrane permeability facilitating membrane re-use. Foaming and emulsifying properties of the UF fractions will be compared with those of original insect protein concentrates and those obtained using a classical chemical fractionation method, such as acidic solubilization.

## 2. Materials and Methods

### 2.1. Materials

Partially defatted black soldier fly powder (BSF) was kindly provided by Hexafly (Ireland). Mealworm (*Tenebrio molitor*) powder (TM) was purchased from Kreca (The Netherlands). Sodium hydroxide pellets (NaOH, Chem-Lab NV, Zedelgem, Belgium) and hydrochloric acid (37–38% HCL, J. T. Baker, Schwerte, Germany) were used for both BSF and TM protein extraction. For defatting of TM powder, 2-methyltetrahydorfuran (EMPLURA, Darmstadt, Germany) was used. Phosphate buffer (pH 7) was prepared using sodium phosphate dibasic heptahydrate (HNa_2_O_4_P7H_2_O, ACROS, Barcelona, Spain) and sodium phosphate monobasic monohydrate (H_2_NaO_4_PH_2_O, ACROS, Barcelona, Spain). Acetic buffer (pH 5) was prepared with sodium acetate (Sigma-Aldrich, St. Louis, MO, USA) and acetic acid (96%, Panreac, Barcelona, Spain). 

### 2.2. Defatting of Mealworm (TM) Powder 

The defatting of TM powder was carried out as per the method provided by Wang et al. [21] with a slight modification. In brief, TM powder (50 g) was mixed with the 2-methyltetrahydrofuran at a ratio of 1:5 (*w*/*v*) and the mixture was stirred at 600 rpm on a magnetic stirrer (AGIMATIC-S 7000242, J.P. SELECTA, Barcelona, Spain) for 1 h. Then, the mixture was allowed to stand and settle until complete phase separation, and the solvent layer was decanted. The remaining precipitated TM powder was mixed again with 2-methyltetrahydrofuran at a ratio of 1:5 (*w*/*v*) and the separation–extraction process was carried out twice more. At the end of the defatting process, the remaining solvent in the precipitate, after the solvent layer was decanted, was evaporated overnight by air drying in a fume hood. Dried, defatted TM powder was then used for protein extraction.

### 2.3. Black Soldier Fly (BSF) and Mealworm (TM) Protein Concentrates

The protein extraction process for BSF and mealworm to obtain the protein concentrates was based on that of Wang et al. [5] with some modifications. The defatted TM or partially defatted BSF powder (30 g) was mixed with 0.25 M NaOH solution at a ratio 1:5 (*w*/*v*) separately and the mixture was heated to 40 °C for one hour with constant agitation at 400 rpm on a magnetic stirrer (RCT ST, IKA, Königswinter, Germany). The mixture was centrifuged (Meditronic 7000599, J.P. SELECTA, Barcelona, Spain) at 3200× *g* for 15 min and the supernatant was separated to continue the protein extraction. In BSF supernatant samples the lipid fraction on the top layer was carefully separated. The pH (Accumet AE150, Fisher Scientific, Singapore) of the supernatant was adjusted to reach a value between 4.0–4.2 by adding 37% hydrochloric acid followed by centrifugation at 2233× *g* for 15 min. After centrifugation, the pellets were collected in aluminum petri dishes and were kept at −60 °C. The entire process was repeated twice with the pellets that remained after the first centrifugation step. The pellets from the three centrifugation processes were combined and freeze-dried (LYOQUEST-85 PLUS, Telstar, Barcelona, Spain) for 24 h at 0.2 mbar with the plates heated to 20 °C. Freeze-dried protein concentrates were stored in the fridge until further use. 

### 2.4. Membranes

Ultrafiltration experiments were performed using regenerated cellulose membranes from Millipore (PLTK04310 and lot no. C2EB73191) with a molecular weight cut-off of 30 kDa, filter diameter of 44.5 mm and filtration area of 13.4 cm^2^. All membranes were conditioned as per the guidelines provided by manufacturer before use. Briefly, the membrane disc was carefully removed from the protective package and placed glossy-side-down in a beaker of Milli-Q water for 20 min. This process was repeated three times in total. The clean water flux was determined and recorded at 5 × 10^4^ Pa and 500 rpm. Then, the conditioned membrane disc was stored in wet conditions at 4 °C until further use. 

### 2.5. Ultrafiltration of Black Soldier Fly (BSF) and Mealworm (TM) Protein Concentrates

**Figure 1:**
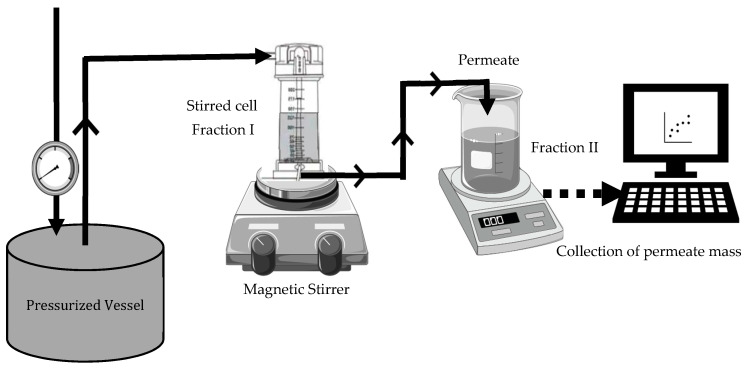
Schematic diagram for ultrafiltration set-up used for fractionated BSFPC and TMPC solutions.

Fractionation of black soldier fly protein concentrate, BSFPC, and mealworm protein concentrate, TMPC, was performed using an experimental apparatus that consisted of a stirred cell (UFSC05001, Amicon 8050, Bedford, MA, USA) connected to a stainless-steel pressurized vessel (Aisi 316L, Advantec MFS, Dublin, CA, USA) containing a protein solution (Figure 1). All the experiments were carried out at room temperature (20–22 °C), with a stirring speed of 500 rpm and constant transmembrane pressure of 5 × 10^4^ Pa. 

Freshly prepared 10 or 7.5 g/L protein concentrate solution (BSFPC or TMPC) was loaded into the apparatus. In brief, a protein solution was prepared with 20 g of freeze-dried protein concentrate powder in 400 mL of phosphate buffer (pH 7) and stirred for 2 h while adjusting the pH to 7 with 4 M sodium hydroxide. The solution was then stored at 4 °C overnight. Subsequently, the solution was centrifuged (Meditronic 7000599, J.P. SELECTA, Barcelona, Spain) twice at 2860× *g* for 15 min. The supernatant was used to prepare 10 or 7.5 g/L protein concentrate solution for the UF run. A new or cleaned 30 kDa membrane was used for each experiment. The permeate was collected in a reservoir placed on an electronic balance (AND FX-3000i, A&D company Ltd, Tokyo, Japan) interfaced to a computer to collect and record time and mass data every 30 s throughout ultrafiltration, which lasted for 90–120 min. Moreover, 500 µL of permeate was collected at 15 min intervals during UF. Each run was performed in duplicate. The fraction which was concentrated inside the stirred cell was collected (referred as Fraction I hereinafter) and the permeate was also collected (referred as Fraction II hereinafter) separately and stored at 4 °C until further use. Further, after each run the membrane was unmounted and cleaned with 0.1 M sodium hydroxide solution for 30 min followed by 100 ppm sodium hypochlorite solution for 30 min. Then, the cleaned membranes were measured for the recovery of clean water flux, and for membranes which were not above the 85% of recovery for clean water flux the cleaning procedure was repeated until the accepted level of flux recovery was reached. The apparatus, without the membrane, was cleaned with 1% sodium hydroxide solution followed by a rinse with distilled water. 

#### Permeate Flux

Permeate flux (L·m^−2^·h^−1^) was calculated for each ultrafiltration run according to Equation (1).

(1) $$\mathrm{Permeate}\;\mathrm{flux}\;\left(J\right)=\frac{\Delta W\;\left(g\right)}{\Delta t\;\left(h\right)\times A\;\left(m^{2}\right)}$$

where Δ*W* is the change in mass at the *i^th^* point, Δ*t* is the change in time at the *i^th^* point, and A is the filtration area of the membrane. Water density was taken to convert from mass to volume.

### 2.6. Black Soldier Fly Protein Concentrate and Mealworm Protein Concentrate Isolation by Acidic Solubilization

**Figure 2:**
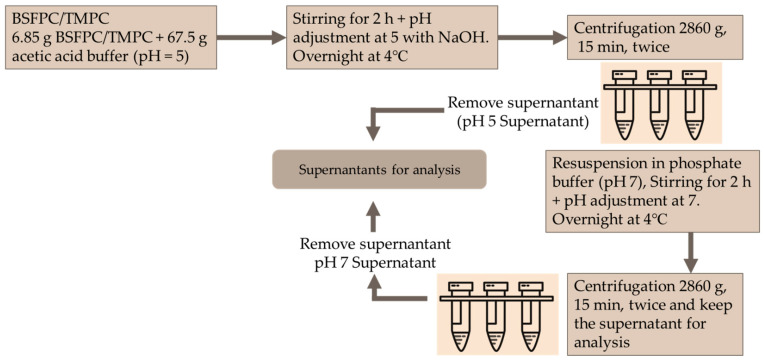
Schematic diagram for acidic solubilization of BSFPC and TMPC solutions.

BSFPC and TMPC solutions were fractioned using the pH shifting method (Figure 2). Briefly, a 10 g/L protein solution was prepared by mixing 6.75 g of BSFPC or TMPC protein powder with 67.5 g of acetic acid buffer (pH 5) and stirred for 2 h while adjusting the pH to 5 with 4 M sodium hydroxide. The mixture was stored at 4 °C for overnight. Subsequently, the mixture was centrifuged (Meditronic 7000599, J.P. SELECTA, Barcelona, Spain) twice at 2860× *g* for 15 min. The supernatant was collected and stored at 4 °C for further analysis. The pellet was re-suspended with the same amount of phosphate buffer (pH 7) for 2 h while adjusting pH to 7 with 4 M sodium hydroxide. The resuspended mixture was stored at 4 °C overnight. The supernatant was collected after centrifugation (Meditronic 7000599, J.P. SELECTA, Barcelona, Spain) two times at 2860× *g* for 15 min. The collected supernatant was stored at 4 °C for further analysis. As controls, both BSFPC and TMPC solutions were prepared in phosphate buffer (pH 7) as mentioned in Section 2.4. 

### 2.7. Characterization of the Black Soldier Fly and Mealworm Protein Fractions

#### 2.7.1. Protein Content

Protein fractions from both ultrafiltration and acidic solubilization processes were analyzed to determine the total soluble protein content using a colorimetric method with a Pierce™ bicinchoninic acid (BCA) protein assay kit (Thermo Scientific, Rockford, IL, USA). BCA assay protein quantification uses bovine serum albumin (BSA) as a standard, and the results are expressed in BSA-equivalent value. To be noted is that black soldier fly and mealworm concentrations that are BSA eq g/L are provided hereinafter as g/L for simplicity.

#### 2.7.2. Surface Charge (ζ Potential) 

Zeta potential of protein fractions from ultrafiltration was measured using Zetasizer Nano-ZS (Malvern Instruments, Worcestershire, UK). Samples were diluted 10 times by phosphate buffer (pH 7). 

#### 2.7.3. Molecular Weight Distribution of Protein Fractions (SDS-PAGE)

The molecular weight distribution of the protein fractions from ultrafiltration and acidic solubilization was determined by sodium dodecyl sulfate–polyacrylamide gel electrophoresis (SDS-PAGE) under reducing conditions [22]. Samples (10 µL) at a concentration of 2 mg/mL and/or 12 mg/mL were loaded to a 4–20% Mini-PROTEAN^®^ TGX™ precast gel (Bio-Rad, Hercules, CA, USA). Electrophoresis was run at 80 V for 100 min. Gel was stained with Coomassie blue G250 (Bio-Rad, Hercules, CA, USA) solution for 45 min followed by destaining for 2–3 h. The molecular weight marker (Precision plus protein dual color standard, Bio-Rad, Hercules, CA, USA) with bands varied from 250 kDa to 10 kDa was used as the standard (Bio-Rad, Hercules, CA, USA).

### 2.8. Techno-Functional Properties of the Black Soldier Fly and Mealworm Protein Fractions

#### 2.8.1. Foaming Capacity and Foam Stability

The foaming properties were analyzed according to the literature [5,21] with minor modifications. A sample of 10 mL of 1 g/L protein solution prepared with BSF or TM protein fractions and appropriate buffer (5 mM, pH 7 or pH 5) were placed in a 50 mL plastic tube and subjected to vigorous rotor-stator homogenization (Ultra Turrax T18 digital, IKA, Königswinter, Germany) at 12,000 rpm for 2 min. The height of the foam layer after 10 s and 120 min was recorded. Foaming capacity (FC) and foam stability (FS) (from experiments run in duplicate) were calculated using Equations (2) and (3), respectively [5]:

(2) $$\%FC=\frac{H_{t}}{H_{0}}\times 100$$

(3) $$\%FS=\frac{FC_{120}}{FC_{0}}\times 100$$

where $H_{0}$ is the initial height of protein solution in the tube, $H_{t}$ is the height of generated foam after agitation, $FC_{0}$ is the initial foaming capacity (after 10 s) and $FC_{120}$ is the one obtained after 120 min.

#### 2.8.2. Emulsifying Activity (EA)

EA was always evaluated at 1 g/L protein concentration using the method described by Purschke et al. [2]. Briefly, 5 mL of protein solution and 5 mL of sunflower oil were homogenized in a beaker using Ultra Turrax T18 digital disperser at 11,000 rpm for 30 s. An aliquot of 9 mL of the emulsion was transferred into a 15 mL scaled tube and centrifuged at 3250× *g* for 20 min at room temperature. Duplicates were performed for each sample. The height of the emulsified layer was noted, and the emulsifying activity was calculated using Equation (4) [5]:

(4) $$\%EA=\frac{H_{EL}}{H_{S}}\times 100$$

where $H_{EL}$ is the height of emulsified layer and $H_{S}$ is the total height of solution in the tube.

### 2.9. UF Membrane Characterization

Morphology of cross-sectional area of a new-conditioned membrane was assessed using a field emission scanning electron microscope (FESEM, Scios™ 2**,** FEI Company Ltd., Hillsboro, OR, USA). Membrane samples were cut using cryogenic scissors and then were fixed on carbon supports to examine under FESEM. 

### 2.10. Statistical Analysis

Results are expressed as mean ± standard deviation. Data from techno-functional properties and zeta potential were analyzed using the general linear model (GLM) at a 95% confidence interval using Minitab 19™ (Minitab Inc., Chicago, IL, USA). 

## 3. Results and Discussion

### 3.1. Fractionation of BSFPC and TMPC by Ultrafiltration: Effect of the initial Protein Concentration on Permeate Fluxes, Protein Transmission, and Membrane Performance Recovery

**Figure 3:**
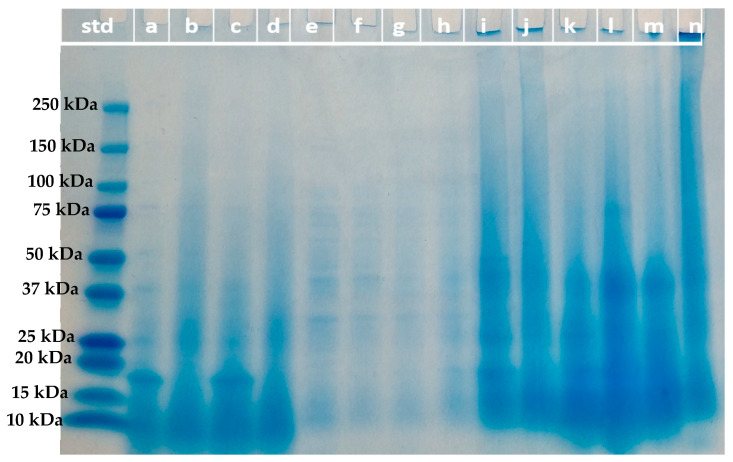
SDS-PAGE results: Sample from the left to right; lane std: Marker; lane a: 10 g/L TMPC FI (2 mg/mL); lane b: 10 g/L BSFPC FI (2 mg/mL); lane c: 7.5 g/L TMPC FI (2 mg/mL); lane d: 7.5 g/L BSFPC FI (2 mg/mL); lane e: 10 g/L TMPC FII (12 mg/mL); lane f: 10 g/L BSFPC FII (12 mg/mL); lane g: 7.5 g/L TMPC FII (12 mg/mL); lane h: 7.5 g/L BSFPC FII (12 mg/mL); lane i: Original BSFPC (2 mg/mL); lane j: Original TMPC (2 mg/mL); lane k: TMPC pH 5 supernatant (12 mg/mL); lane l: TMPC pH 7 supernatant (12 mg/mL); lane m: BSFPC pH 5 supernatant (12 mg/mL); lane n: BSFPC pH 7 supernatant (2 mg/mL). FI: Fraction I; FII: Fraction II.

As mentioned in the introduction, there is no previous literature on the fractionation of insect proteins by membrane processes. The two insect species selected for the study have been reported to have different protein size distributions when obtained under similar extraction conditions to the ones used in our study. BSF presented two major bands at 14.3 and 80.5 kDa [17] while TM showed a different distribution of the protein fractions, with a major fraction between 13–40 kDa and another fraction between 67–250 kDa [17]. According to the SDS-PAGE analysis of the protein concentrates used in the present study (Figure 3, samples i and j, respectively), TMPC presents fractions between 10–15 kDa, a fraction close to 20 kDa, and another one at about 25 kDa. Moreover, a fraction between 37–50 kDa is also identified, as well as the presence of proteins of higher molecular weight. For BSFPC, there is a clear fraction below 20 kDa, a fraction between 20–30 kDa, and proteins of higher molecular weight are also present but with no distinctive bands.

**Figure 4:**
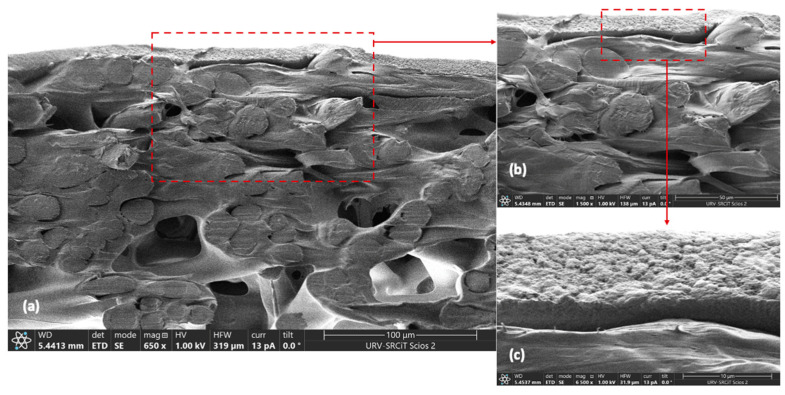
FESEM images of the clean regenerated cellulose acetate membrane at different magnifications (**a**–**c**).

Based on the literature and the results obtained from the SDS-PAGE analysis (Figure 3), regenerated cellulose acetate membranes of 30 kDa were selected for insect protein fractionation in concentration mode. The membrane used has a clear asymmetric structure (Figure 4a) with two layers, a dense active layer facing the feed (9–14 μm, Figure 4c) over a loose fiber layer acting as a support. The pure water flux of the membrane at the fractionation conditions (5 × 10^4^ Pa and 500 rpm) was 328 ± 11 L h^−1^ m^−2^.

Since fractionation was assessed to enhance foaming and emulsifying properties of the insect proteins, a permeate with enough protein concentration to test for those techno-functional properties without any further processing was required. To do so, preliminary tests were performed (data not shown) and a feed protein concentration of 10 g/L resulted in permeate concentrations of about 1–2 g/L for both insect species, which is in the range that can be tested using the methodologies presented in Section 2.7.1 and Section 2.7.2. Moreover, based on those preliminary tests, it was clear that with a 10 g/L protein solution in the feed, an increase in pressure up to 1.5 × 10^5^ Pa did not increase the permeate flux. These findings indicated that fractionation under those experimental conditions was beyond the limiting flux. Therefore, to increase the permeate flux and study the impact on protein transmission, a decrease in the feed concentration to 7.5 g/L was assessed for both insect proteins, maintaining the rest of the experimental conditions as unchanged.

**Figure 5:**
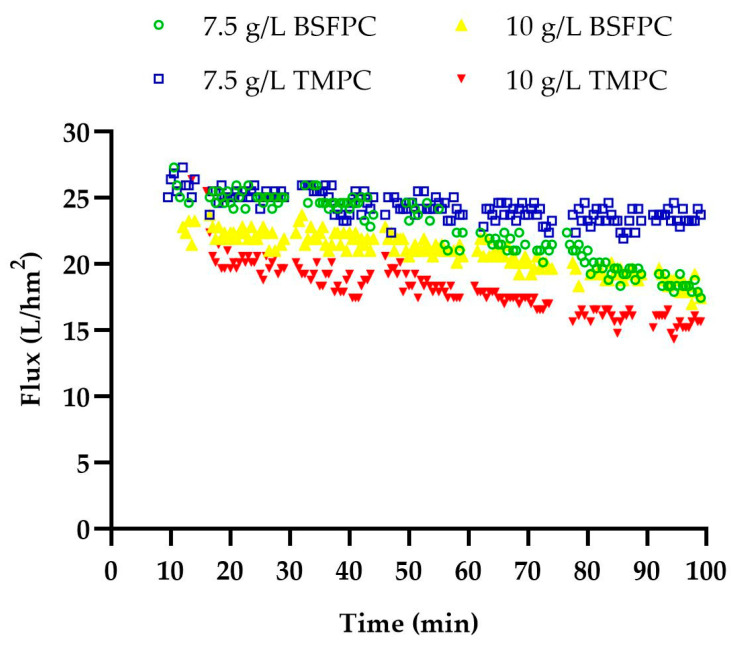
Permeate flux evolution during filtration of 10 and 7.5 g/L BSFPC and TMPC using a 30 kDa regenerated cellulose acetate membrane. Each point represents the average of two replicates. Standard deviation has not been included for clarity purposes.

**Figure 6:**
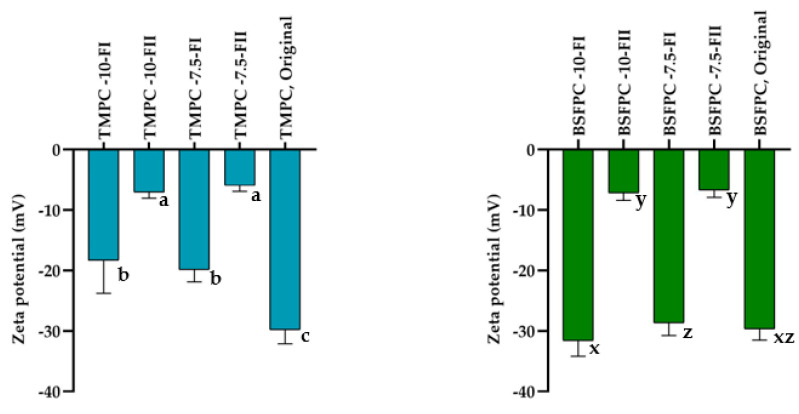
ζ potential of fractions from ultrafiltration and original BSFPC and TMPC protein solutions. Error bars show standard deviation.

Figure 5 shows permeate flux evolution during ultrafiltration of 10 and 7.5 g/L BSFPC and TMPC solutions under concentration mode at 5 × 10^4^ Pa and 500 rpm. From the permeate flux evolution, a different behavior can be observed for BSFPC and TMPC when changing the initial protein concentration. While there was an increase in the permeate flux for TMPC when decreasing the concentration from 10 to 7.5 g/L, the decrease in the initial protein concentration did not affect the permeate flux evolution for BSFPC. Therefore, there is a clear difference in the behavior of the two proteins during ultrafiltration that can be attributed either to the different interactions between the protein and the membrane or to the protein gel layer formed on top of the membrane. As the ζ potential of the two protein solutions is quite similar (Figure 6), the differences observed during protein fractionation with the 30 kDa membrane should be attributed to the different composition of the insect protein concentrates resulting in different gel layers on top of the membrane. The permeate fluxes obtained after 100 min of filtration can be found in Table 1. From these values it can be seen that, for the highest protein concentration, BSFPC had a higher permeate flux after 100 min of filtration than TMPC, but it did not increase when the initial concentration decreased to 7.5 g/L. Conversely, TMPC—even though it has a lower permeate flux for the highest protein concentration than BSFPC—presented an increase in permeate flux of about 50% when decreasing the initial protein concentration to 7.5 g/L.

**Figure 7:**
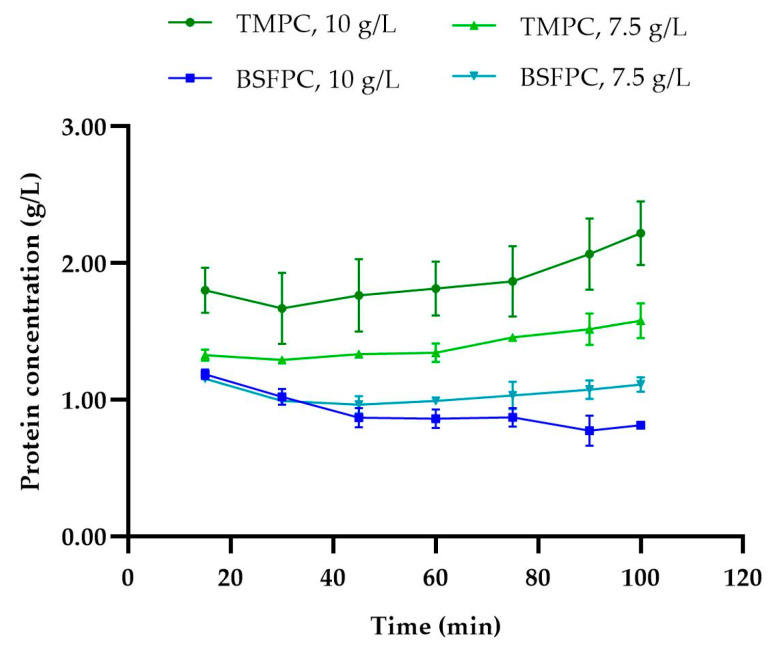
Protein concentration evolution at the outlet of the membrane during filtration of 10 and 7.5 g/L solutions of BSFPC and TMPC. Error bars show standard deviation.

Samples of the permeate were collected every 15 min to follow protein concentration in the permeate during filtration, since the protein concentration obtained in the permeate at the end of the process provides an average value. Figure 7 shows the evolution of protein concentration at the outlet of the membrane during the ultrafiltration process. As can be seen, there is a different trend depending on the insect protein as in the case of permeate fluxes. While for BSFPC the protein concentration measured throughout filtration was kept almost constant for an initial feed concentration of 7.5 g/L or slightly decreased for 10 g/L initial concentration, TMPC protein concentration slightly increased throughout the filtration regardless of the initial protein concentration. This trend also points out a different behavior of the proteins during ultrafiltration which should be explained by the different composition and nature of the proteins and is beyond the scope of this study. Moreover, the concentrations of the two fractions (Fraction I and Fraction II) obtained after UF are presented in Table 2, together with the labels used to identify each fraction hereafter. For BSFPC, the concentration of Fraction I was about 15 g/L regardless of the initial concentration while, for TMPC, Fraction I concentration increased from about 15 g/L to about 27 g/L with an increase in the feed concentration. 

Since there are no reports on fractionation of insect proteins by membrane ultrafiltration, the possibility of membrane re-use after applying the cleaning protocols suggested by the manufacturer was important to investigate. After the filtration, each membrane was cleaned following the procedure detailed in Section 2.4, and water flux measured. It was found that water flux could be restored after cleaning to more than 85% of the initial flux, with an average value of 294 ± 9 L h^−1^ m^−2^. Therefore, the cleaning protocols suggested by the membrane manufacturer can be applied after insect protein filtration, and membranes could be re-used within the experimental tests performed in the present study.

### 3.2. Emulsifying Activity of the BSFPC and TMPC Fractions 

**Figure 8:**
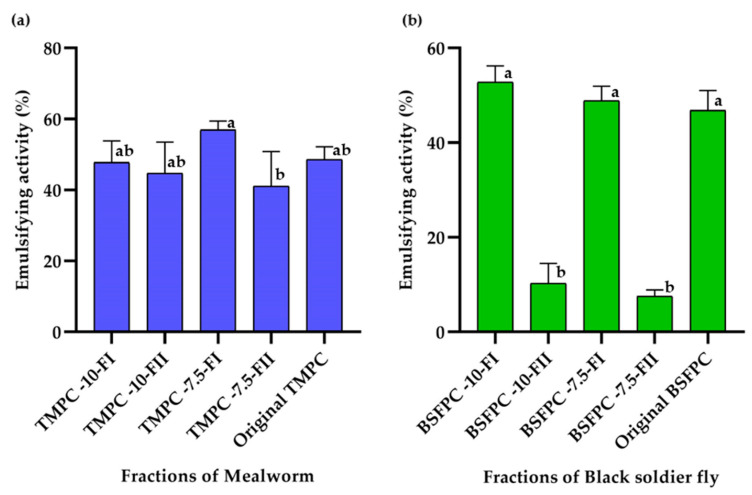
Emulsifying activity (%) of UF fractions from (**a**) mealworm and (**b**) black soldier fly. Means that do not share a letter are significantly different. Error bars show standard deviation.

Among the uses of insect proteins in feed and food applications, replacement of dairy proteins as emulsifiers has been highlighted [5,9]. To do so, emulsifying properties have to be studied and enhanced if possible. The literature shows that BSF and TM proteins can be used to stabilize emulsions [5,13], but there are still no reports on the possibility of using membrane separation to obtain protein fractions with enhanced interfacial properties. From the UF fractionation trials, two protein fractions were obtained for each insect species and initial feed concentration. To obtain comparable results on emulsifying activity for all the protein fractions, the protein concentration was adjusted to 1 g/L for all the samples, including the original protein concentrates (BSFPC and TMPC), which served as a control value. Figure 8 plots the emulsifying activity, calculated as explained in Section 2.7.2, for all the protein fractions obtained after ultrafiltration of BSFPC and TMPC with two different initial feed concentrations. First of all, it is interesting to point out that EA of the samples corresponding to Fraction I (concentrate) is maintained in the same range as the original protein concentrate, regardless of the protein type and initial feed concentration (Figure 8a). These values are consistent with those reported in the literature for the emulsifying capacity of mealworm obtained by isoelectric point precipitation [14] and show a good ability of the two insect proteins to stabilize emulsions [6]. However, it has to be noted that, since the emulsifying properties reported in the literature come from fractions obtained with different extraction techniques, the comparison can be misleading.

Regarding the permeate fractions (Fraction II), we can observe a different EA depending on the type of insect protein. The EA of the permeate fraction of TMPC obtained with a 10 g/L protein feed solution has a similar value to the original TMPC. When the feed concentration was decreased to 7.5 g/L, the permeate fraction had an EA value in the same range as the original TMPC but was significantly lower than the EA of the concentrate fraction (Figure 8a). Conversely, the permeate protein fractions from BSFPC had a significatively lower EA than the original protein and concentrated protein fractions, regardless of the initial feed concentration (Figure 8b). Emulsifying activity depends on the protein concentration—which was maintained as constant in all the tests—and on factors such as surface hydrophobicity of the proteins, conformation stability, and charge [23]. Our results suggest that the proteins able to reach the permeate for BSFPC have a much lower ability to interact with the oil phase and absorb on the oil side. As for the effect of surface charge, BSFPC permeate fractions have low values of surface charge (ranged from −6.73 to −7.19 mV, Figure 6) regardless of the initial feed concentration, but this cannot be the only factor, since the surface charge of the TMPC permeate fractions is in the same range (Figure 6), and needs to be investigated further.

The SDS-PAGE characterization performed for all the samples can provide some information regarding the impact of the different protein fractions in their ability to stabilize oil–water interfaces. For TMPC concentrates (Fraction I) SDS-PAGE results show a fraction of proteins of about 17 kDa (Figure 3a,c), while other fractions of 25, 37, 50, and 75 kD are still present. Since the 17 kDa protein fraction is the only one not found in the TMPC permeate fractions (Figure 3e,g), it could indicate that the proteins in the 17 kDa range do not have a key role in the emulsion stabilization process.

Regarding fractionation of BSFPC, the SDS-PAGE results of Fraction I (Figure 3b,d) show proteins with molecular weight below 15 kDa to be more concentrated as well as a fraction between 25–35 kDa. The EA values of 7.5 and 10 g/L BSFPC Fraction I were in the same range than the original black soldier fly protein concentrate. However, the EA values of the permeates have an extremely low value of EA that cannot be explained only by the protein fractions present, since the SDS-PAGE (Figure 3f,h) does not show different bands than the permeates obtained from TMPC. 

### 3.3. Foaming Properties of the BSFPC and TMPC Fractions 

**Figure 9:**
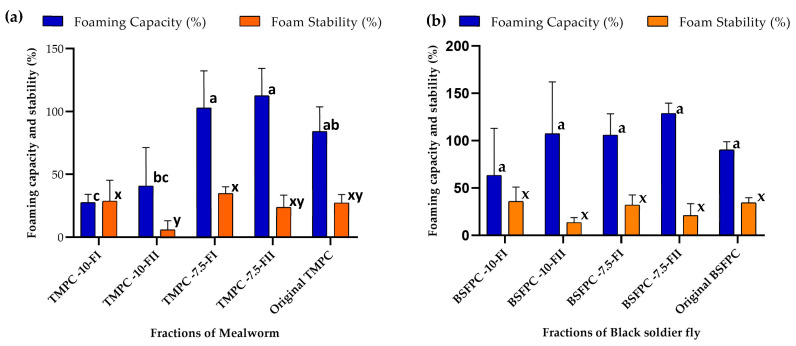
Foaming capacity (%) and foam stability (%) of fractions from (**a**) mealworm (**b**) black soldier fly compared to the original protein concentrates. Means that do not share a letter are significantly different. Error bars show standard deviation.

Foaming properties (foaming capacity and foam stability) will indicate the extent of absorption at the air–liquid interfaces, and are important for several food production processes, such as those of ice-cream or beverages [23]. The FC and FS values for the fractions obtained by UF are presented in Figure 9. The results indicate that, for TMPC, the concentrate and permeate fractions obtained using a 10 g/L initial concentration have a lower FC than the original protein, while the ones obtained with an initial feed concentration of 7.5 g/L exhibited comparable FC values to the original protein (Figure 9a). The foam stability of all the TMPC fractions obtained from UF was similar to the original or lower, as in the case of the permeate fraction using a feed concentration of 10 g/L (Figure 9a). All in all, the potential of the fractions obtained with UF for foam stabilization can be tuned depending on the original protein concentration, as that will influence the proteins able to pass through the membrane. 

For the fractions obtained with BSFPC, both FC and FS were maintained in the same range as the original protein (Figure 9b) for the two initial feed concentrations studied (*p* > 0.05). Again, the comparison with the values reported in the literature can be misleading since there are slight variations in the determination method and also in the extraction conditions. The values reported by Mshayisa et al., 2022, [20] indicate an enhancement of the foaming properties after defatting the insect power. Since all the protein concentrates employed in the present study were already defatted and obtained using extraction at isoelectric point precipitation (pH 4), the foaming properties of the fractions maintain the potential of the original and are not enhanced. Nonetheless, the values obtained for the original and protein fractions suggest potential for use in the food industry to replace dairy proteins.

### 3.4. Effect of Acidic Solubilization Fractionation on Emulsifying and Foaming Properties

**Figure 10:**
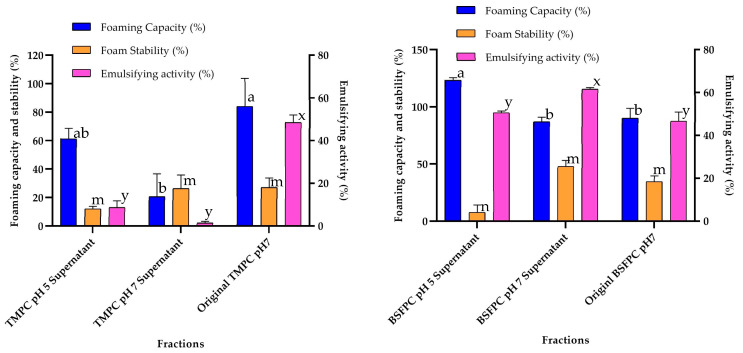
Foaming capacity (%), foam stability (%), and emulsifying activity (%) of TMPC and BSFPC fractions obtained by acidic solubilization. Error bars show standard deviation.

It has already been described that protein recovery using acid or alkaline solutions can be an alternative way to obtain fractions with improved properties. Therefore, the acid solubilization strategy explained in Section 2.5 was applied to assess whether fractions with enhanced techno-functional properties could be obtained. Since the protein fractions fed to the UF module were obtained after solubilization with a pH 7 buffer, the solubilization process shown in Figure 2 was considered. The BSFPC and TMPC were first solubilized with a pH 5 buffer and the resulting protein solutions (BSFPC pH5 and TMPC pH5) were tested for emulsifying and foaming properties and molecular weight distribution with SDS Page. A further solubilization step with a pH 7 buffer of the remaining precipitate (Figure 2), allowed to obtain another protein fraction (BSFPC pH7 and TMPC pH7), also tested for techno functional properties, Figure 10, and molecular weight distribution, Figure 3. 

The acidic solubilization protocol applied to TMPC produced two protein fractions with lower EA than the original protein, as was also the case for FC and FS (Figure 10a). However, in the case of BSFPC, the acidic solubilization led to two fractions with improved properties compared to the original protein. On the one hand, the protein fraction obtained after solubilization at pH 5 has higher FC than the original protein, even though the foam stability was lower (Figure 10b), and on the other, the fraction resulting after solubilization of the remaining pellets at pH 7 had enhanced emulsifying activity compared to the original protein. Unfortunately, the results about the molecular weight distribution (Figure 3, k-n) do not provide relevant information on the protein molecular weight fractions that could be responsible for the loss of interfacial properties in the case of TMPC or the enhancement in the case of BSFPC. The only remarkable difference from Figure 3 obtained by comparing fractions j (original BSFPC) and n (BSFPC supernatant pH 7), both loaded at the same concentration, is that for the BSFPC supernatant pH 7—besides the protein fractions present in the original protein—fractions of higher molecular weight distribution are present that could be responsible for the improvement in the EA and FC. 

## 4. Conclusions

This study showcases for the first time the feasibility of ultrafiltration to fractionate mealworm and black soldier fly protein concentrates. When ultrafiltration is carried out using a 30 kDa regenerated cellulose acetate membrane in concentration mode, initial protein concentrations of 10 and 7.5 g/L led to permeate fluxes ten times lower than that of water under the same transmembrane pressure (5 × 10^4^ Pa). Permeate flux decreased during filtration for both proteins and concentrations; however, a decrease in the initial protein concentration only had a positive impact on the flux for TMPC. This, and the fact that protein concentration at the outlet of the stirred cell increased during TMPC filtration, point out the effect of the protein composition on the performance of the process. The existing membrane-cleaning protocol was proven successful in recovering the permeate water flux after filtration of both proteins regardless of the initial concentration, enabling membrane re-use. Even though the emulsifying activity and the foaming properties of the fractions obtained by UF were not enhanced, the values are of interest for industrial applications to replace dairy proteins. Moreover, acidic solubilization has allowed the preparation of fractions with enhanced emulsifying activity and foaming capacity for black soldier fly. Combining acidic and/or alkaline solubilization with UF to obtain insect protein fractions can definitively be explored. This study opens the door to membrane technology for insect protein fractionation, which has not been studied so far and has already provided useful solutions for other animal and plant proteins. 

## Figures and Tables

**Table 1:** Permeate fluxes for BFSPC and TMPC at the end of filtration with a 30 kDa regenerated cellulose acetate membrane at 5 × 10^4^ Pa and 500 rpm.

System	Permeate Flux (L/h m^2^)
10 g/L BSFPC	18.32 ± 0.73
7.5 g/L BSFPC	18.42 ± 0.53
10 g/L TMPC	15.58 ± 0.59
7.5 g/L TMPC	23.64 ± 0.48

**Table 2:** Soluble protein content of fractions obtained from ultrafiltration. Fraction I corresponds to the concentrate fraction, while Fraction II corresponds to the permeate fraction.

Fractionation Method	Insect Protein	Fraction	Label	Protein Content (g/L)
Ultrafiltration	7.5 g/L BSFPC	Fraction I	BSFPC-7.5-FI	15.09 ± 0.24
	7.5 g/L TMPC	Fraction I	TMPC-7.5-FI	15.15 ± 0.29
	7.5 g/L BSFPC	Fraction II	BSFPC-7.5-FII	1.14 ± 0.01
	7.5 g/L TMPC	Fraction II	TMPC-7.5-FI	1.41 ± 0.01
	10 g/L BSFPC	Fraction I	BSFPC-10-FI	15.88 ± 2.22
	10 g/L TMPC	Fraction I	TMPC-10-FI	26.76 ± 1.80
	10 g/L BSFPC	Fraction II	BSFPC-10-FII	0.91 ± 0.03
	10 g/L TMPC	Fraction II	TMPC-10-FII	1.80 ± 0.26

## Data Availability

Not applicable.
